# Pre-Analytical and Clinical Validation of a Dried Blood Spot Assay for Asymmetric Dimethylarginine and L-Arginine

**DOI:** 10.3390/jcm9041072

**Published:** 2020-04-09

**Authors:** Juliane Hannemann, Thore I. Roskam, Ina Eilermann, Patricia Siques, Julio Brito, Rainer Böger

**Affiliations:** 1 Institute of Clinical Pharmacology and Toxicology, University Medical Center Hamburg-Eppendorf, 20246 Hamburg, Germany; thore.roskam@gmx.de (T.I.R.); ina-eilermann@web.de (I.E.); boeger@uke.de (R.B.); 2 Institute of Health Studies, Universidad Arturo Prat, Iquique 1100000, Chile; psiques@tie.cl (P.S.); jbritor@tie.cl (J.B.)

**Keywords:** ADMA, L-arginine, risk factor, biomarker, chronic-intermittent hypoxia, ELISA

## Abstract

Asymmetric dimethylarginine (ADMA) inhibits nitric oxide (NO) synthesis. It is a risk marker for cardiovascular events and mortality in patients with cardiometabolic diseases and in population-based studies. Plasma or serum analysis of ADMA may be hampered by pre-analytical sample handling. We validated a dried blood spot (DBS) assay for ADMA and L-arginine and show here that this assay has excellent variabilities and reproducibilities. Filter paper is impregnated with the arginase inhibitor nor-NOHA (N^ω^-hydroxy-nor-Arginine) to avoid L-arginine degradation. Clinical validation of this DBS assay confirms elevated ADMA concentration in hemodialysis patients as compared to healthy controls, higher ADMA concentrations in men versus women, and elevated L-arginine concentration in subjects supplemented with L-arginine. The DBS assay was used in a cohort study involving 100 primarily healthy subjects in the Andean region to assess the impact of chronic intermittent hypoxia on ADMA and L-arginine; ADMA DBS concentration at sea level was prospectively associated with pulmonary hypertension after six months of exposure to 3500 m. In a cohort of 753 individuals, L-arginine/ADMA ratio significantly decreased with increasing number of traditional cardiovascular risk factors. Analysis of ADMA and L-arginine in DBS is a reliable and reproducible method for quantitation of these markers in field studies.

## 1. Introduction

Asymmetric dimethylarginine (ADMA) is an endogenous, modified amino acid that was first described in 1970 by Kakimoto and Akazawa [1]. It is formed during the transfer of two methyl groups onto L-arginine moieties of proteins by the activity of a family of enzymes named protein arginine N-methyltransferases (PRMTs) [2]. ADMA has gained much research interest after the discovery that it acts as a competitive inhibitor of nitric oxide (NO) synthesis [3,4]. We and others have characterized ADMA as a marker of major adverse cardiovascular events and mortality in a vast number of prospective clinical studies including patients with a broad range of total cardiovascular risk. These studies have been reviewed elsewhere [5]. As examples, patients undergoing hemodialysis treatment have a highly increased rate of cardiovascular morbidity and mortality, which relates to their high ADMA levels [6]. ADMA is also a prospective marker of total mortality in the general population, as evidenced in the Framingham Offspring cohort [7] and in the Gothenburg women study [8]. Through its molecular mechanism of action, the concentrations of both, ADMA and L-arginine are worth considering in order to assess the true availability of substrate for NO synthase [9]. Indeed, the L-arginine/ADMA ratio turned out to be useful as an independent marker of total mortality [7].

A vast range of analytical methods has been presented during the past decade for quantification of ADMA and L-arginine, ranging from HPLC [10] to LC-MS/MS [11] and ELISA [12]. The differences and characteristics of each of these analytical methods have been summarized by our group before [13]. All of these methods, however, are hampered by the need of blood centrifugation, separation of plasma, and storage and transportation in the frozen state. Sometimes, in field studies, there is a lack of equipment for pre-analytical processing of samples, and the length of storage for customs clearance often hampers fast intercontinental transport on dry ice. Analysis of samples that have undergone multiple freeze-thaw cycles delivers unreliable results, specifically for L-arginine [14,15]. For use in epidemiological and interventional field studies and for routine testing in remote areas, a versatile, robust, and cheap method to secure samples for ADMA and L-Arginine analysis is desirable. Dried blood spots have proven to be a method fulfilling these requirements for sample preparation in neonatal screening [16], testing for cystic fibrosis [17], pancreatic function testing [18], and in disease diagnosis in older adults [19]. We have developed a dried blood spot assay that allows for the analysis of both ADMA and L-arginine, and we report here the results of the pre-analytical and clinical validation of this assay.

## 2. Materials and Methods

### 2.1. Assay Development and Variabilities

Capillary blood from one fingertip was taken by a safety lancet (Sarstedt, Nürnbrecht, Germany) and dripped onto certified filter paper with six marked circles of 7 mm diameter each (Sartorius Stedim, Göttingen, Germany). Six drops of capillary blood were dripped onto the filter paper in a manner that the blood spots were visible on the reverse side of the filter paper to ensure that the filter paper was sufficiently soaked with blood. The filter paper was allowed to dry before further processing. For each individual, three dried blood spots of 5 mm diameter were punched out and used for analysis.

To determine intra-assay variability, five filter cards were filled with six blood spots each from each of five healthy subjects at one single time point. Three spots per filter card were punched out of each filter card and measured in one single assay run.

To assess inter-assay variability, another set of five filter cards were filled with six blood spots each from each of five healthy subjects at one single time point. Blood spots from one filter card per subject were punched out and measured in one assay; this procedure was repeated on five consecutive days.

Biological day-to-day variability of measurements within subjects were determined by filling one filter card per day with blood from three healthy volunteers on five different days. The blood spots were analyzed on the day of blood sample collection.

### 2.2. Analysis of ADMA and L-Arginine in Dried Blood Spots

Three dried blood spots were used for each measurement. Blood spots from each subject were collected in reaction tubes and incubated with 300 µL of elution buffer (main active component, hydrochloric acid; pH = 1.0) on an orbital shaker for 20 min. Elution capacity of this procedure was characterized by recovery rates of 96% for ADMA and 62% for L-arginine. Samples were centrifuged at 3000 g, and two aliquots of 75 µL each were used for analysis after acylation (60 min, room temperature). Analysis of ADMA and L-arginine was performed using commercially available ELISA assays (DLD Diagnostika, Hamburg, Germany) according to the manufacturer’s instructions. The cross-reactivities of the antibodies used in the two ELISA parts are given as follows: ADMA ELISA, <0.01% with L-arginine, 1.41% with SDMA; L-arginine ELISA, 0.36% with ADMA, 0.72% with SDMA. The ADMA ELISA has previously been validated by our group [12].

### 2.3. Analysis of ADMA and L-Arginine in Plasma Samples

Analysis of ADMA and L-arginine in plasma was performed by liquid chromatography–tandem mass spectrometry (LC-MS/MS) using a validated and published method [11]. Briefly, 25 μL of plasma was diluted with stable isotope-labeled internal standards. Proteins were precipitated with methanol; the guanidine compounds were converted to their butyl esters and analyzed by LC–MS/MS (Varian 1200 MS, Agilent Technologies, Santa Clara, CA, USA). Quantification was performed by calculation of peak area ratios and calibration with known concentrations of analytes in dialyzed EDTA plasma. The analytical range of the method was validated from 0.05 to 4 μmol/L for ADMA and SDMA, respectively, and from 0.5 to 250 μmol/L for L-arginine, and mean coefficients of variation were ≤ 5% for all analytes. All other laboratory values were measured using routine clinical laboratory methods.

### 2.4. Influence of Storage Conditions on Assay Results

To determine the influence of temperature and humidity on assay results, 5 filter cards from 4 healthy volunteers were stored at room temperature (20 °C) or at 35 °C for one month. At each temperature, filter cards were exposed either to dry conditions (average laboratory air humidity; 40–60% air humidity) or to humid conditions (95% air humidity).

In a second experimental setup, filter cards were stored at room temperature under dry (*n* = 4) or humid conditions (*n* = 8) for 24 h. A subgroup of four filter cards in the high humidity exposure group had been impregnated with nor-NOHA (N^ω^-hydroxy-nor-Arginine), an inhibitor of arginase (400 mmol/L, 25 µL per spot), before filling with blood.

In a final experimental setup, four filter cards from each of five healthy volunteers were impregnated with nor-NOHA, dried, and filled with blood. They were either analyzed on the same day or stored at room temperature for up to 307 days. One filter card per subject was analyzed after 21, 76, and 307 days of storage, respectively.

### 2.5. ADMA and L-Arginine Concentrations in Dried Blood Spots versus Plasma

Blood was drawn from 17 healthy volunteers and from 20 hemodialysis patients. Two samples per study participant were taken: One sample was withdrawn from a forearm vein using vacutainers with EDTA sodium to generate plasma after centrifugation (2000× *g*, 10 min, 20 °C). Plasma was stored at −20 °C until analysis of ADMA and L-arginine by LC-MS/MS. Another sample from each study participant was collected onto filter cards impregnated with nor-NOHA after poking one fingertip with a safety lancet. All plasma samples and all dried blood spots were analyzed in the same assays, respectively, within five days after sample collection.

### 2.6. Clinical Validation of the Dried Blood Spot Assay: Healthy Individuals Exposed to Chronic Intermittent Hypoxia

In this study, we analyzed the association of dried blood spot ADMA with mean pulmonary arterial pressure (mPAP), and we compared the changes in ADMA and L-arginine from baseline to six months in dried blood spots and plasma, respectively. Dried blood spots were collected from 100 healthy male Chilean subjects who were exposed to six months of chronic intermittent hypoxia at high altitude. We measured ADMA and L-arginine from the dried blood spots that had been taken at baseline (sea level) and after six months of exposure to a regimen of 5 days at high altitude (3500 m) alternating with 2 days at sea level. A subgroup of 44 study participants underwent echocardiographic evaluation at six months to estimate mean pulmonary arterial pressure (mPAP) as described before [20]. The analysis of plasma ADMA in this cohort and the suitability of ADMA as a predictor of high altitude-associated pulmonary arterial hypertension have been published by us before [20]. This study was approved by the Ethics Committee of the University Arturo Prat, Iquique, Chile, all participant gave their consent to participate.

### 2.7. Clinical Validation of the Dried Blood Spot Assay: Assay Results in Individuals with a Broad Range of Cardiovascular Risk

We retrospectively analyzed the assay results from 753 individuals who had ADMA and L-arginine levels measured in dried blood spots for a variety of motivations, ranging from an individual history of cardiovascular disease to the presence of cardiovascular risk factors, to a health-conscious lifestyle, and to intake of a dietary supplement containing L-arginine. Each individual filled a short, structured questionnaire asking for medical history, presence of cardiovascular risk factors (i.e., hypertension, hypercholesterolemia, smoking, and diabetes), and some anthropometric variables such as systolic and diastolic blood pressure, total cholesterol, LDL and HDL cholesterol, triglyceride, and creatinine serum levels if known to the individual. All participants agreed to evaluation of their data; the aggregated statistical analyses for this study were performed in a strictly anonymized manner.

### 2.8. Statistical Analyses

Statistical analyses were performed using SPSS (version 25; IBM Corporation, Armonk, NY, USA) and GraphPad Prism (version 6.01; GraphPad Software Inc., San Diego, CA, USA). All variables were tested for normal distribution using the Kolmogorov–Smirnov test. Differences between groups were tested for significance by using either the nonparametric Mann–Whitney *U* test for two groups or the Kruskal–Wallis analysis of variance for more than two groups. The association of dried blood spot ADMA with mPAP was calculated using linear regression. Receiver-operated curve (ROC) analyses were constructed for this association to assess the optimal cut-off value for ADMA. Data are presented as mean with standard deviation. For all tests, *p* < 0.05 was considered significant.

## 3. Results

### 3.1. Precision and Accuracy of the Dried Blood Spot Assay

Measurement of ADMA and L-arginine in dried blood spots from 5 technical replicates of each of 5 individuals showed intra-assay variabilities of 6.7% (95% CI: 2.7%; 10.7%) for ADMA and 6.5% (0.8%; 12.2%) for L-arginine. Inter-assay variabilities were 10.4% (6.9%; 13.9%) for ADMA and 11.5% (8.4%; 14.6%) for L-arginine (Table 1). To determine intra-individual stability of measurements over time, samples of three individuals were taken on five consecutive days and measured immediately after drying. Variation was 8.5% (4.3%; 12.7%) for ADMA and 17.8% (9.3%; 26.4%) for L-arginine.

### 3.2. Effects of Pre-Analytical Sample Treatment

**Figure 1:**
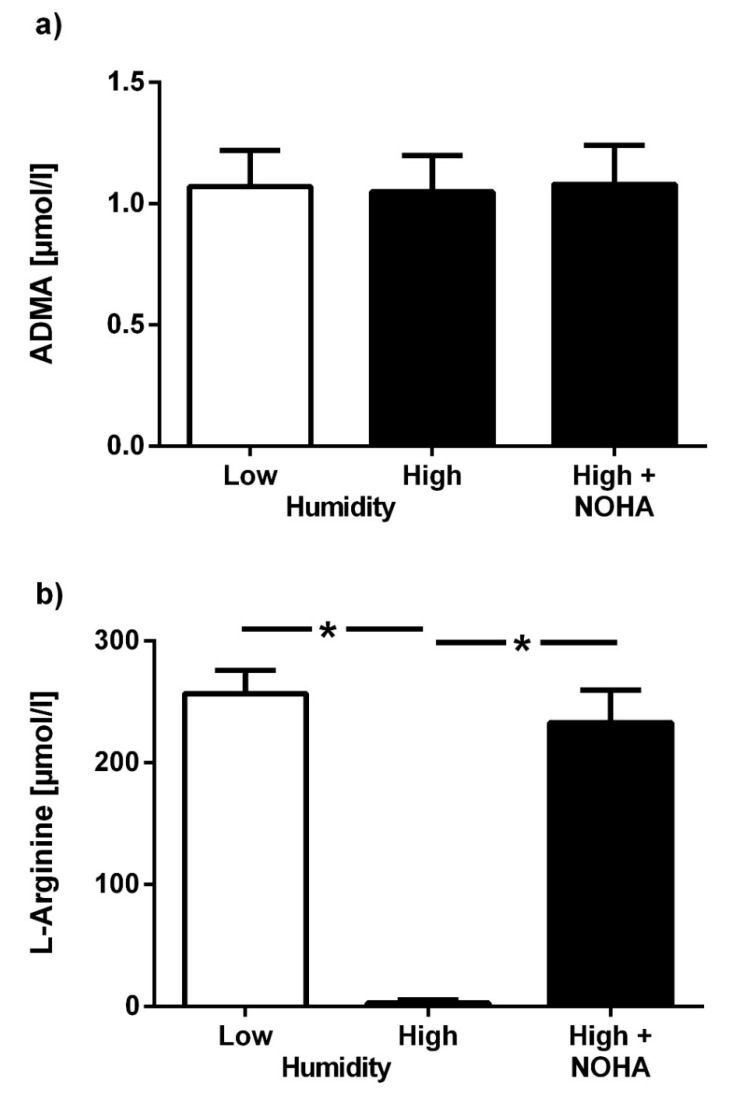
Effect of air humidity on dried blood spot assay results. Concentrations of asymmetric dimethylarginine (ADMA) (**a**) and L-arginine (**b**) in untreated dried blood spots subjected to low (40–60%) or high air humidity (95%), as compared to dried blood spots pre-treated with the arginase inhibitor, nor-NOHA, at high air humidity. Data are presented as mean ± SD; * *p* < 0.05 between groups.

To assess the influence of storage conditions on test results, the effects of ambient temperature and air humidity on untreated filter cards were assessed. Mean ADMA in dried blood spot assays stored at room temperature under dry conditions for 24 h was 1.07 ± 0.15 µmol/L; mean ADMA in dried blood spots stored at 95% humidity for 24 h was 1.05 ± 0.15 µmol/L (*p* = not significant (n.s.)); Figure 1a). The respective mean concentrations for L-arginine were 256.8 ± 19.4 µmol/L and 5.9 ± 2.6 µmol/L in dry and humid conditions, respectively (*p* < 0.001; Figure 1b). L-arginine concentration in samples that were treated with the arginase inhibitor, nor-NOHA, and kept under humid conditions for 24 h was 233.1 ± 26.8 µmol/L (*p* < 0.001 vs. untreated samples in humid conditions, *p* = n.s. vs. low humidity; Figure 1b). ADMA concentration was not significantly affected by treatment of the filter cards with nor-NOHA.

**Figure 2:**
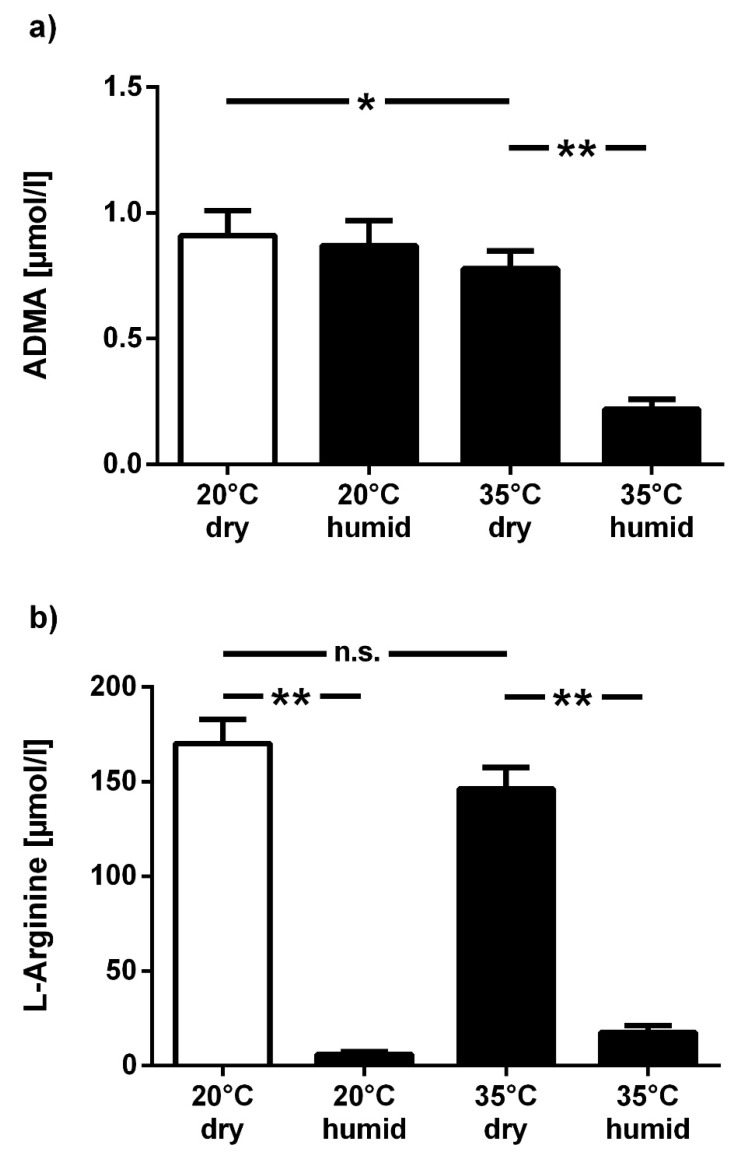
Combined effect of temperature and humidity on dried blood spot assay results. Filter cards were exposed to 20 °C or 35 °C, either at low (40–60%) or high air humidity (95%) for one month, and ADMA (**a**) and L-arginine (**b**) were measured from the dried blood spots. Data are presented as mean ± SD; * *p* < 0.05, ** *p* < 0.01 between groups; n.s.: not significant.

To further analyze the combined effects of temperature and humidity on test results, samples were kept at 20 °C or 35 °C, either at dry or humid conditions. ADMA concentration was lower in filter cards that were kept at 35 °C, with an additive effect of high humidity (−14.6% and −75.5%, respectively; *p* < 0.05 and *p* < 0.01 vs. 20 °C and dry conditions) (Figure 2a), while L-arginine concentration dropped substantially in humid conditions, both at 20 °C (−96.5%) and 35°C (−89.7%; *p* < 0.01 for both vs. 20 °C and dry conditions) (Figure 2b).

Comparison of filter cards filled with 50 µL or 100 µL of whole blood revealed no significant difference in ADMA and L-arginine concentration, provided that the filter paper was completely soaked by the blood (ADMA, 0.86 ± 0.01 and 0.93 ± 0.01 µmol/L; L-arginine, 82.2 ± 5.5 and 74.0 ± 11.8 µmol/L, respectively; both *p* = n.s.).

Daily exposure to UV light during one month did not significantly modify ADMA and L-arginine concentrations.

**Figure 3:**
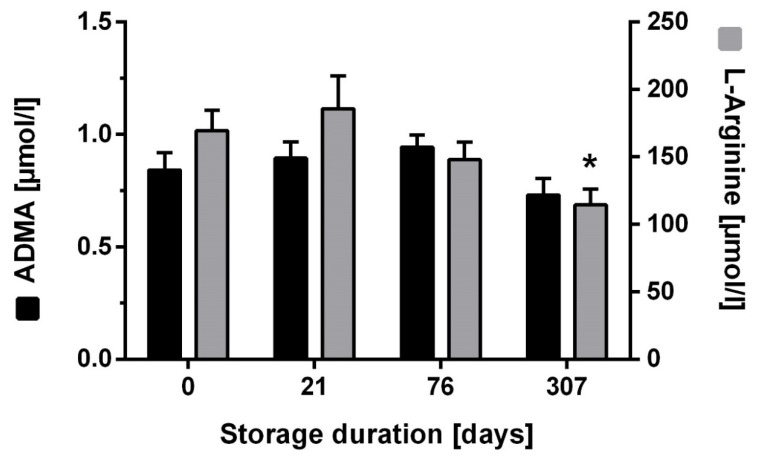
Effect of long-term storage at room temperature on dried blood spot assay results. ADMA and L-arginine were measured from dried blood spots on filter cards that had been pretreated with nor-NOHA and stored for up to 307 days at room temperature. Data are presented as mean ± SD; * *p* < 0.05 vs. day 0.

Long-term storage of filter cards treated with nor-NOHA for up to 307 days at room temperature with low humidity showed stability of ADMA concentration over the whole period (−10.5 (+10.5; −31.6) % at 307 days; Figure 3). By contrast, L-arginine concentration was only stable for up to 72 days and showed a significant drop at 307 days (−30.5 (+4.7; −65.7) %; *p* < 0.05; Figure 3).

### 3.3. ADMA and L-Arginine Concentrations in Dried Blood Spots versus Plasma

**Figure 4:**
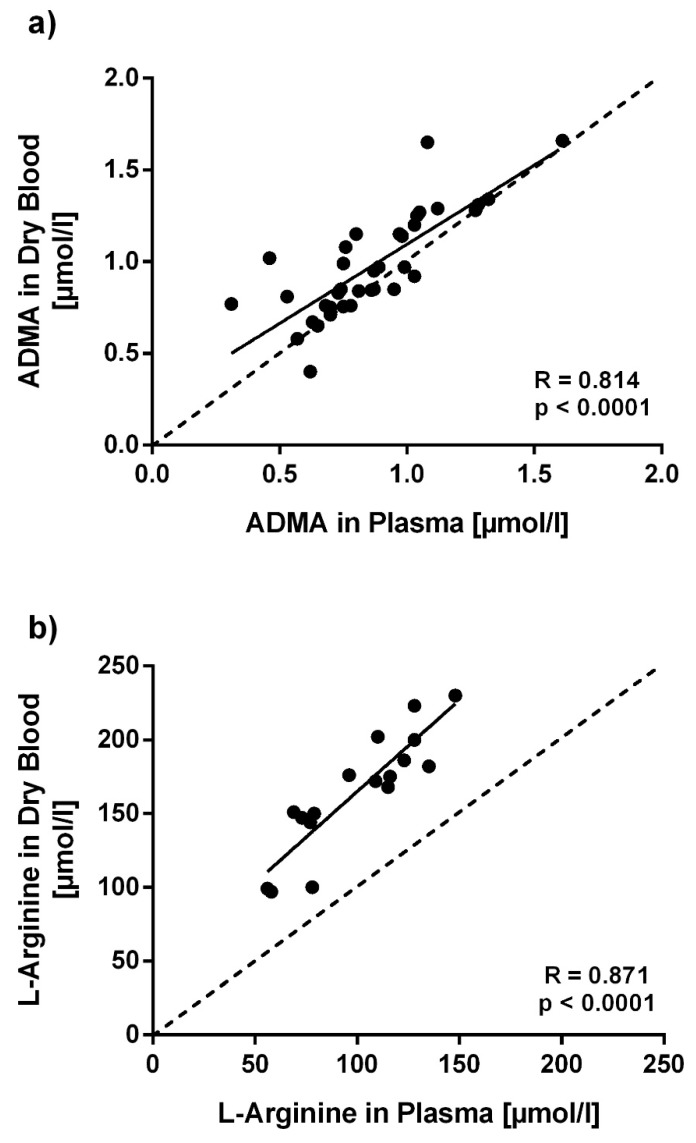
Correlation of ADMA and L-arginine concentrations measured in dried blood spots and in plasma. ADMA (**a**) and L-arginine (**b**) were measured from dried blood spots and plasma samples collected from the same subjects at the same day. Each dot marks one subject’s assay results. The dotted line marks the line of equivalence between both assays. R values represent Pearson’s correlation coefficients.

ADMA concentration using the dried blood spot assay was significantly higher in hemodialysis patients than in healthy volunteers (1.11 ± 0.05 µmol/L vs. 0.78 ± 0.05 µmol/L; *p* < 0.001). When ADMA levels measured with the dried blood spot assay were compared to plasma ADMA levels measured by LC-MS/MS in these two groups, ADMA levels measured with the dried blood spot assay were significantly higher than those measured in plasma (0.86 ± 0.26 µmol/L vs. 0.98 ± 0.28 µmol/L; *p* < 0.01). A significant linear correlation of assay results was found (R = 0.814; *p* < 0.001; Figure 4a). L-arginine concentration measured with the dried blood spot assay also had significantly higher results than L-arginine levels measured by LC-MS/MS in plasma (99.9 ± 28.7 µmol/L vs. 164.8 ± 40.0 µmol/L; *p* < 0.01); nonetheless, results obtained with both methods also showed a significant linear correlation (R = 0.871; *p* < 0.001; Figure 4b).

### 3.4. Clinical Validation of the Dried Blood Spot Assay: Healthy Individuals Exposed to Chronic Intermittent Hypoxia

**Figure 5:**
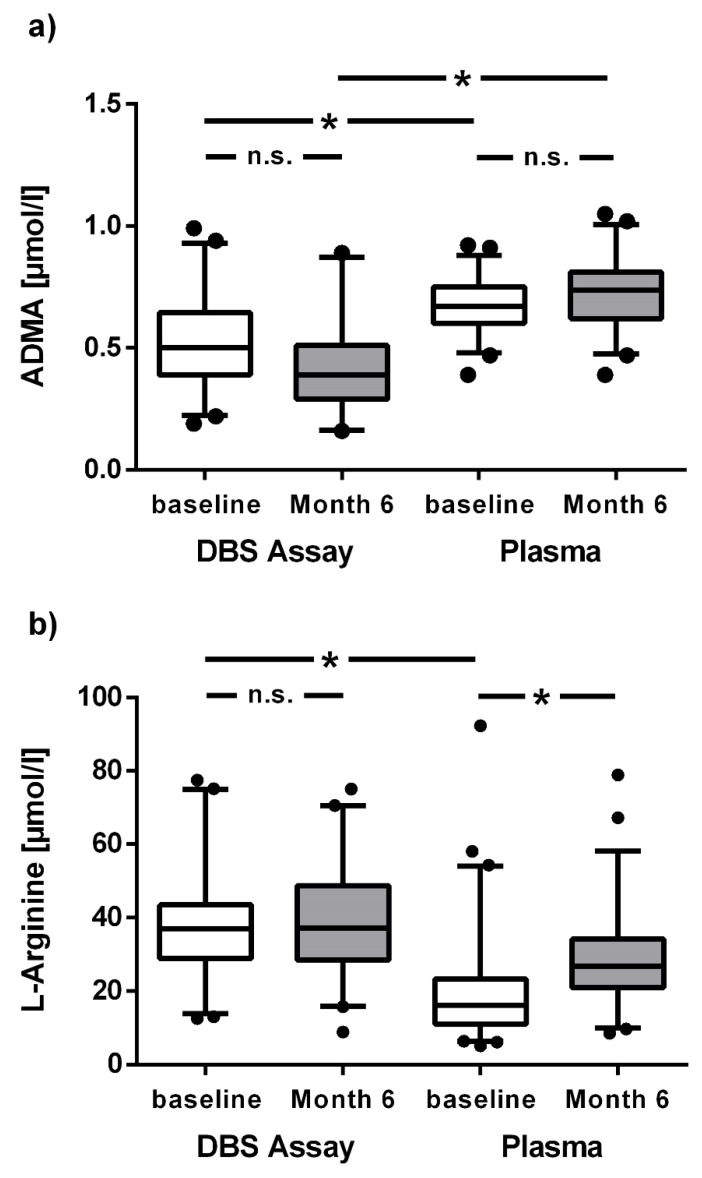
Box plots representing concentrations of ADMA and L-arginine at baseline and after 6 months of chronic intermittent hypoxia (CIH). ADMA (**a**) and L-arginine (**b**) were measured from dried blood spots and plasma in individuals exposed to six months of chronic intermittent hypoxia (CIH). * *p* <0.05 between groups.

**Figure 6:**
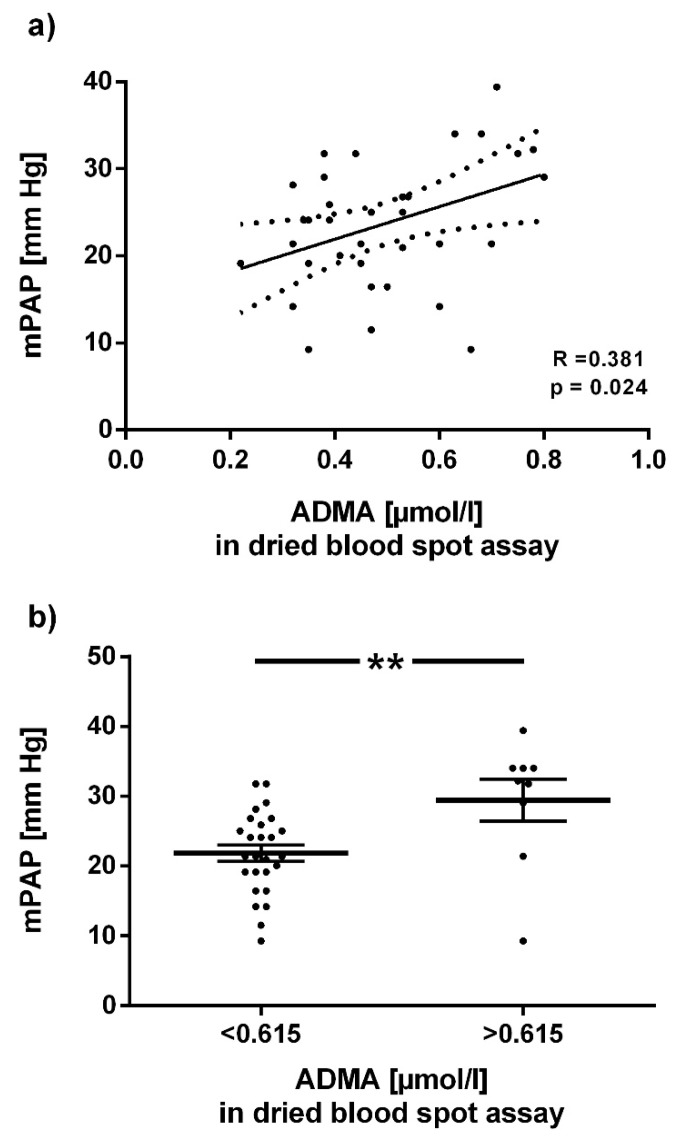
Association of baseline ADMA concentration in dried blood spots with mean pulmonary arterial pressure after 6 months of CIH. (**a**) Linear regression analysis of dried blood spot ADMA concentration at baseline with mean pulmonary arterial pressure (mPAP) after six months of exposure to chronic intermittent hypoxia (CIH). (**b**) Mean pulmonary arterial pressure (mPAP) at month 6 of CIH exposure in individuals with baseline dried blood spot ADMA concentration below or above o.615 µmol/L, the optimal cut-off for discrimination of the two groups in ROC analysis. Horizontal lines indicate mean ± SD; each dot represents one individual. ** *p* < 0.01 between groups.

ADMA and L-arginine concentrations were measured in a cohort of 100 Chilean healthy males before and at the end of 6 months of exposure to chronic intermittent hypoxia; the assay results were compared with L-arginine plasma concentrations at both time points. ADMA concentrations in the dried blood spot assay were significantly lower than those in plasma at baseline and after 6 months (Figure 5a). By contrast, L-arginine concentration at baseline was significantly higher in the dried blood spot assay than in plasma; after 6 months this difference was attenuated (Figure 5b). Baseline ADMA concentration in the dried blood spot assay was positively associated with mean pulmonary arterial pressure after 6 months (R = 0.382; *p* = 0.02; Figure 6a). In ROC analysis, the optimal cut-off ADMA concentration in dried blood spot assay to discriminate individuals with high vs. low mPAP at 6 months was 0.615 µmol/L; individuals with ADMA > 0.615 µmol/L had significantly higher mPAP (*p* = 0.005; Figure 6b).

### 3.5. Clinical Validation of the Dried Blood Spot Assay: Assay Results in Individuals with a Broad Range of Cardiovascular Risk

We included 753 individuals (422 women, 331 men) aged 57.8 ± 13.5 years in this analysis. Mean ADMA concentration in dried blood spots was 0.88 ± 0.25 µmol/L, mean L-arginine concentration was 130.6 ± 97.5 µmol/L, resulting in a mean L-arginine/ADMA ratio of 152.8 ± 112.6.

**Figure 7:**
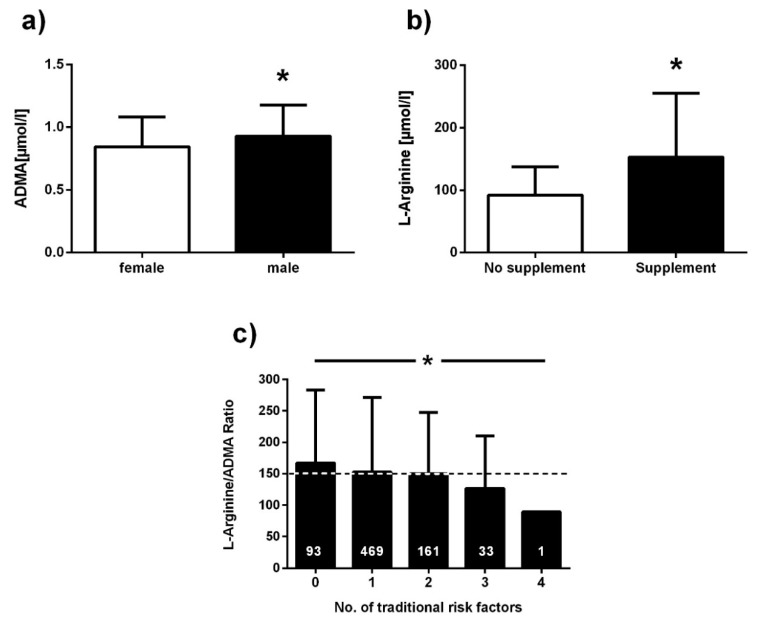
Clinical validation of dried blood spot ADMA and L-arginine. (**a**) ADMA concentration in dried blood spots in male and female subjects; (**b**) L-arginine concentration in dried blood spots in individuals taking L-arginine supplements or not; (**c**) L-arginine/ADMA ratio according to number of traditional risk factors present. Data are presented as mean ± SD; * *p* < 0.05 between groups in (**a**) and (**b**); * *p* < 0.05 for trend across risk factor groups in (**c**).

**Figure 8:**
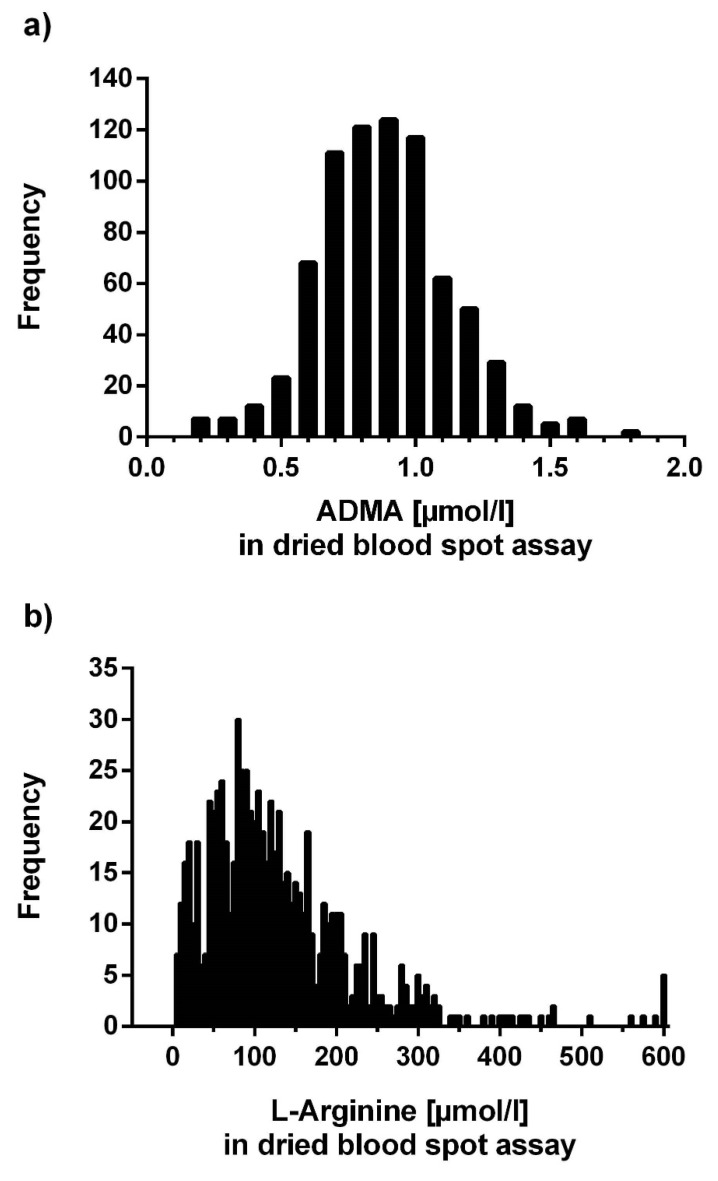
Distribution of ADMA concentrations (**a**) and L-arginine concentrations (**b**) in dried blood spots.

Mean ADMA was higher in men than in women (0.93 ± 0.25 µmol/L vs. 0.84 ± 0.24 µmol/L; *p* < 0.0001; Figure 7a). Individuals who had experienced myocardial infarction tended to have higher ADMA than those without myocardial infarction (0.92 ± 0.23 µmol/L, *n* = 70; vs. 0.88 ± 0.25 µmol/L, *n* = 648; *p* = 0.150). Smokers had slightly lower mean ADMA concentration than nonsmokers and ex-smokers (0.87 ± 0.24 µmol/L vs. 0.88 ± 0.25 µmol/L; *p* = n.s.). L-Arginine concentration in dried blood spots was significantly higher in individuals who used oral L-arginine supplements than in those who did not (153.2 ± 102.0 µmol/L vs. 124.7 ± 95.5 µmol/L; *p* = 0.001; Figure 7b). With an increasing number of traditional cardiovascular risk factors present, there was a trend towards higher ADMA and an inverse trend towards lower L-arginine, which resulted in a significantly lower L-arginine/ADMA ratio with increasing number of risk factors (*p* < 0.05; Figure 7c). Figure 8 displays the distribution of ADMA and L-arginine concentrations in dried blood spots in the population studied.

## 4. Discussion

The present study shows that ADMA and L-arginine can be measured in dried blood spots by ELISA with good accuracy and reproducibility. The dried blood spot assay is robust against pre-analytical ambient factors except high humidity and very high temperature, and samples can be stored for long periods of time before analysis. We have applied the assay in various human populations and found differences in ADMA and L-arginine levels corresponding to those previously reported using other analytical methods by us and others.

ADMA has been extensively characterized as a competitive inhibitor of NO synthesis; elevated ADMA concentrations have been shown to be involved in the development of endothelial dysfunction [21], hypertension [22], acute coronary syndrome [23], and atherosclerotic vascular disease [5]. As ADMA acts by displacing L-arginine, the natural substrate of NOS, from the catalytic site, the ratio of the concentrations of ADMA and L-arginine is important to assess NOS substrate availability in vivo [9]. Indeed, both ADMA and the L-arginine/ADMA ratio have been shown to be associated with the risk of major adverse cardiovascular events and mortality [7].

Among the various methods that have been developed to enable the measurement of ADMA and L-arginine, HPLC- and LC-MS/MS-based methods have received the most attention. These methods are useful in clinical studies, as LC-MS/MS is regarded as the gold standard for quantitation of small molecules due to the utilization of stable isotope-labeled internal standards that ensure high accuracy and precision of the method [24,25]. Beyond this, immunoassay methods are available, for example an ELISA assay that was validated by our group [10]. ELISA methods have the advantage of not depending on sophisticated, expensive laboratory equipment, which makes these methods more ubiquitously available; however, separate ELISA assays are needed to assess both, ADMA and L-arginine plasma concentrations. In a comparison of analytical methods for ADMA, Schwedhelm reported that ADMA plasma concentrations measured with ELISA are comparable to those obtained with LC-MS/MS, while most HPLC-based methods find slightly lower ADMA levels [13].

All of these previously developed analytical methods are hampered by the fact that fresh or frozen plasma or serum samples are needed. However, the necessary equipment is not always available to ensure timely and accurate separation of plasma or serum from the blood collection tube and instant freezing of unspoiled (i.e., nonhemolytic) samples, specifically in field studies in remote areas. The dried blood spot method that we present here provides a unique tool to allow for quick and easy sample collection and storage under a broad range of ambient conditions except high temperature and air humidity. After elution of the blood from the filter paper, ELISA methodology is used to quantify both, ADMA and L-arginine. These features make the method ideally suitable for field studies, as collection of capillary blood is easy and may therefore be performed by medical support staff. Furthermore, the actual sample processing for analysis can be executed in one central laboratory that collects dried blood spots from many more or less distant locations, thereby limiting the need for analytical laboratory equipment at multiple sites and, at the same time, increasing the number of samples in the central laboratory due to expansion of its reach. In contrast to frozen plasma specimen, shipment of samples is possible by ordinary surface mail without further precautions.

Clinical validation of the dried blood spot method showed elevated ADMA levels in hemodialysis patients as compared to healthy controls. Elevation of plasma dimethylarginine levels in patients with renal failure has been the first clinical observation made since the discovery of ADMA as an inhibitor of NO synthesis [4,26]. Higher ADMA concentration is associated with higher all-cause mortality and cardiovascular event rate in hemodialysis patients [6].

With the dried blood spot method, we were also able to reproduce our recent finding that plasma ADMA concentration increases during chronic exposure to intermittent hypoxia [20], and that baseline ADMA plasma concentration is prospectively associated with the development of high altitude pulmonary arterial hypertension (HAPH). In the present study, we show that the same clinical relationship can also be found when using dried blood spot ADMA levels. The cut-off ADMA level for discrimination of individuals with or without HAPH was slightly different from the plasma cut-off value; however, our validation experiments showed that dried blood spot ADMA levels are higher than the corresponding plasma concentrations.

The differences in ADMA and L-arginine concentrations that we found between the dried blood spots and plasma may relate to the fact that the dried blood spot technique utilizes completely hemolyzed blood. During the drying of blood on the filter paper, hemolysis occurs, so that this method measures the total of plasma and blood cell ADMA and L-arginine. By contrast, liquid sample methods allow for quantification of the plasma (or serum) concentrations of these analytes, not the intracellular amounts within the cytoplasm of blood cells. Indeed, accidental hemolysis may increase variability of the analytical results for L-arginine and ADMA when plasma or serum assays are used. Although Tsikas and co-workers reported that clinically relevant levels of hemolysis do not impair analysis of ADMA [27], it has been known that intracellular concentration is higher than the plasma concentration [28]. For L-arginine, lysis of red blood cells may result in spilling of high L-arginine concentrations into the sample; however, when the sample is left at room temperature for extended periods of time, L-arginine concentration may considerably drop due to the simultaneous release of arginase from the erythrocytes [29]. While hemolysis is inherent to the dried blood spot method, we reduced variabilities by ensuring complete hemolysis for all samples. Impregnation of the filter cards with the arginase inhibitor, nor-NOHA, reliably prevents degradation of L-arginine by arginase during drying of the blood spots on the filter paper and during the elution from the card. The comparatively low variabilities of our assay prove that reproducible results can be obtained.

Using the ADMA and L-arginine dried blood spot method may also be useful for monitoring of nutritional therapy in individuals undertaking oral L-arginine supplementation. We and others have repeatedly shown that oral L-arginine supplementation may help to improve the symptoms of vascular disease in individuals with L-arginine deficiency [30]. L-arginine deficiency, as such, may occur either as absolute L-arginine deficiency, a rare syndrome that is characterized by low circulating L-arginine levels, or as relative L-arginine deficiency. The latter syndrome is much more common, as even in the presence of apparently “normal” L-arginine levels, high ADMA concentration may elicit the need for higher L-arginine concentrations to saturate NOS with substrate [9]. Our clinical validation studies show that the dried blood spot assay may be used to monitor L-arginine concentration during oral supplementation, which is important, as not all L-arginine supplements may provide sufficient L-arginine to elevate the blood levels of this amino acid—a fact that remains unnoticed during unmonitored supplementation.

In conclusion, the dried blood spot assay for the determination of ADMA and L-arginine that we present here is an easy-to-use, highly reproducible, and ubiquitously available method for use in field studies and for clinical monitoring of patients with cardiovascular risk factors or overt disease.

## Figures and Tables

**Table 1:** Variabilities of the ADMA and L-arginine dried blood spot assay.

	Median	95% CI
Intraassay variability	(%)	(%)
ADMA	6.7	2.7–10.7
L-Arginine	6.5	0.8–12.2
Inter-assay variability		
ADMA	10.4	6.9–13.9
L-Arginine	11.5	8.4–14.6
Day-to-day intraindividual variability		
ADMA	8.5	4.3–12.7
L-Arginine	17.8	9.3–26.4

Abbreviations: 95% CI: 95% confidence interval of the median; ADMA: asymmetric dimethylarginine.
